# Signals of drug-related retinal artery occlusion: a multi-country retrospective study from a spontaneous reporting system

**DOI:** 10.3389/fmed.2026.1851758

**Published:** 2026-07-07

**Authors:** Xianfen Cao, Jing Zeng, Shinan Wu, Chenyu Wu, Xiaoping Zhou, Yulun Ou

**Affiliations:** 1 Department of Ophthalmology, The First People’s Hospital of Chenzhou, Hunan, China; 2 Department of Ophthalmology, The First Affiliated Hospital of Jinan University, Guangzhou, China; 3 Ophthalmic Center, The Second Affiliated Hospital of Guangzhou Medical University, Guangzhou, China; 4 Fujian Provincial Key Laboratory of Ophthalmology and Visual Science, Xiamen University Affiliated Xiamen Eye Center, Fujian Engineering and Research Center of Eye Regenerative Medicine, School of Medicine, Eye Institute of Xiamen University, Xiamen University, Xiamen, China; 5 The Affiliated Chenzhou Hospital of University of South China, Hengyang, China

**Keywords:** disproportionality analyses, time-to-onset, FAERS, pharmacovigilance, retinal artery occlusion

## Abstract

**Objective:**

This study aimed to systematically identify systemic drugs associated with retinal artery occlusion (RAO) using real-world data from the US FDA Adverse Event Reporting System (FAERS).

**Methods:**

FAERS reports from January 2004 to December 2024 were analyzed. Disproportionality analyses (ROR, PRR, BCPNN, and MGPS) were applied to detect signals of disproportionate reporting. Drugs were further evaluated based on signal strength and time-to-onset characteristics.

**Results:**

A total of 626 drugs were initially screened, among which 36 drugs were identified as having significant disproportionality signals for RAO. These drugs were categorized into several pharmacological classes, including antineoplastic agents, anti-VEGF agents, reproductive system medications, anesthetics, anti-inflammatory drugs, hormonal agents, and others. Strong signals, as indicated by the BCPNN algorithm, were observed for mepivacaine, brolucizumab, and pegaptanib. Anesthetic agents exhibited the shortest median time to onset.

**Conclusion:**

This study characterizes the reporting patterns and signal strength of RAO across multiple drug classes using pharmacovigilance data. These findings provide real-world evidence to enhance clinical awareness and support safer prescribing practices, while emphasizing the need for further validation in controlled studies.

## Introduction

Retinal artery occlusion (RAO) is a rare but severe retinal vascular disorder that can lead to significant visual impairment (1, 2). It is classified into central RAO (CRAO), branch RAO (BRAO), and cilioretinal artery occlusion, depending on the retinal vessel affected (3). The primary cause of RAO is thromboembolism, typically originating from a large artery or the heart. Additionally, substantial evidence points to a strong association between RAO and systemic vascular events, including stroke, myocardial infarction, and valvular heart disease, while behavioral risk factors such as smoking are also well-established (4–6). Among various risk factors, pharmaceutical agents, though not the most common trigger, are an increasingly significant yet often overlooked cause.

While the systemic management of acute RAO is well-defined, typically involving neurologists with stroke management expertise, no proven specific ocular therapies currently exist (7). Given these therapeutic limitations and the generally poor visual prognosis associated with RAO, a proactive and preventive approach is crucial. Although certain medications, such as oral contraceptives and specific chemotherapeutic agents, have been implicated in RAO through isolated case reports, these observations remain fragmented and lack systematic consolidation (8–10). This highlights the urgent need for a rigorous, comprehensive evaluation of medications associated with RAO risk. This concern is further intensified by the global aging population and the rising prevalence of chronic diseases, which may increase the number of individuals exposed to potential pharmacologic risks.

The FDA Adverse Event Reporting System (FAERS) plays a vital role in post-marketing pharmacovigilance by collecting real-world adverse event reports from healthcare professionals and consumers (11). Although previous studies have explored ocular adverse events associated with certain medications using pharmacovigilance databases, these investigations were generally limited to specific drug classes or individual agents (12). A comprehensive analysis systematically evaluating multiple medications potentially associated with RAO using the FAERS database remains lacking. Our objectives are to identify medications with notable disproportionality signals associated with RAO, characterize the clinical features of these adverse events, and provide insights to enhance risk recognition and inform clinical decision-making.

## Materials and methods

### Data source

**FIGURE 1:**
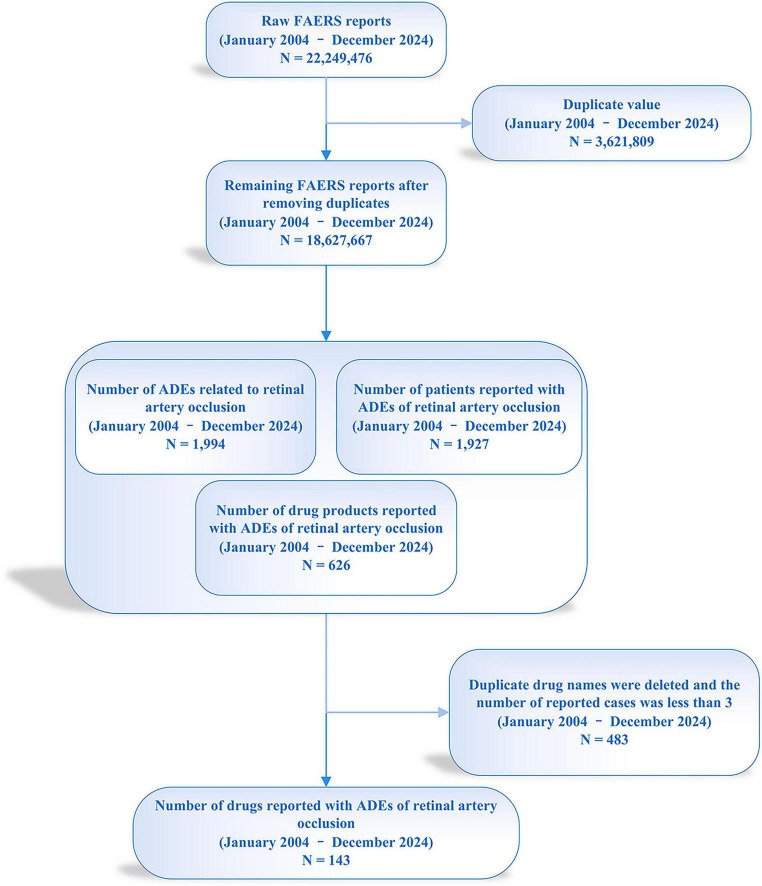
Flowchart of the data cleaning pipeline for drug-related RAO data from the FAERS database.

This study utilized data from the FAERS, part of the FDA’s post-marketing safety surveillance program, which collects adverse event reports from healthcare professionals, consumers, and manufacturers for all marketed drugs and therapeutic biologic products. The analysis covered reports submitted from January 2004 to December 2024, sourced from a publicly accessible database.^1^ Reports submitted by consumers were excluded from the primary analysis to reduce heterogeneity related to reporter type and to increase the clinical consistency of adverse-event coding and drug-event attribution. In FAERS, the term “consumer” refers to a reporter category rather than the presence or absence of personal identification. Anonymous or de-identified reports were retained when the reporter type was not explicitly classified as consumer, because public FAERS data are inherently de-identified and exclusion solely based on anonymity could unnecessarily remove otherwise valid reports. A total of 22,249,476 original entries were recorded in the database from January 2004 to December 2024. After deduplication by primary ID, 18,627,667 entries remained for analysis. Among these, 1,994 reports were identified as RAO-related adverse events, involving 1,927 unique subjects and 626 distinct drugs. Drug names were cross-referenced with the DrugBank database^2^ and standardized to include both generic and brand names (13). Drugs with fewer than three reported cases were excluded, resulting in a final set of 143 drugs associated with RAO. The data cleaning process is illustrated in Figure 1.

### Identification of adverse drug events

Adverse drug events (ADEs) in this analysis were defined using the Medical Dictionary for Regulatory Activities (MedDRA^®^, version 20.0). All adverse events were coded using MedDRA^®^ Preferred Terms (PTs). RAO-associated events were identified using the corresponding Standardized MedDRA Query (SMQ) and restricted to PTs classified within the narrow scope to maximize specificity. The PTs included retinal artery occlusion, central retinal artery occlusion, branch retinal artery occlusion, cilioretinal artery occlusion, and retinal arterial embolism.

### Statistical analysis

Signal detection was performed using four commonly applied disproportionality methods, including the Reporting Odds Ratio (ROR), Proportional Reporting Ratio (PRR), Bayesian Confidence Propagation Neural Network (BCPNN), and Multi-item Gamma Poisson Shrinker (MGPS). These methods are based on fourfold contingency table calculations and are widely used in pharmacovigilance studies to identify potential drug–adverse event associations by comparing the reporting frequency of a target drug–event pair against all other drugs and events in the database.

The thresholds and formulas for the four disproportionality algorithms are detailed in Supplementary Tables 1, 2. Positive signal criteria were defined as follows: (1) ROR: a ≥ 3 with the lower bound of the 95% confidence interval (CI) > 1; (2) PRR: a ≥ 3 with the lower bound of the 95% CI > 1; (3) BCPNN: IC025 > 0; and (4) MGPS: EBGM05 > 2.

To reduce false-positive findings arising from reporting bias or sparse data, a drug was considered a positive signal only when all four algorithms simultaneously met their predefined thresholds. This conservative strategy has been adopted in previous pharmacovigilance studies and enhances the specificity of signal detection by requiring consistency across both frequentist and Bayesian approaches.

Additionally, Time-to-onset (TTO) was defined as the interval between drug initiation (START_DT) and adverse event onset (EVENT_DT) in the FAERS database. Reports with missing or implausible dates were excluded. TTO values above the 99th percentile were removed to reduce the impact of outliers. The remaining data were analyzed using descriptive statistics and cumulative incidence curves across drug classes (14).

Statistical analyses were performed using SPSS (v26.0), GraphPad Prism (v10.1.2), Microsoft Excel, and R (v4.2.2), with a significance threshold of *P* < 0.05. Key R packages used included ggplot2, dplyr, ggrepel, and DescTools.

## Results

### Baseline characteristics

**FIGURE 2:**
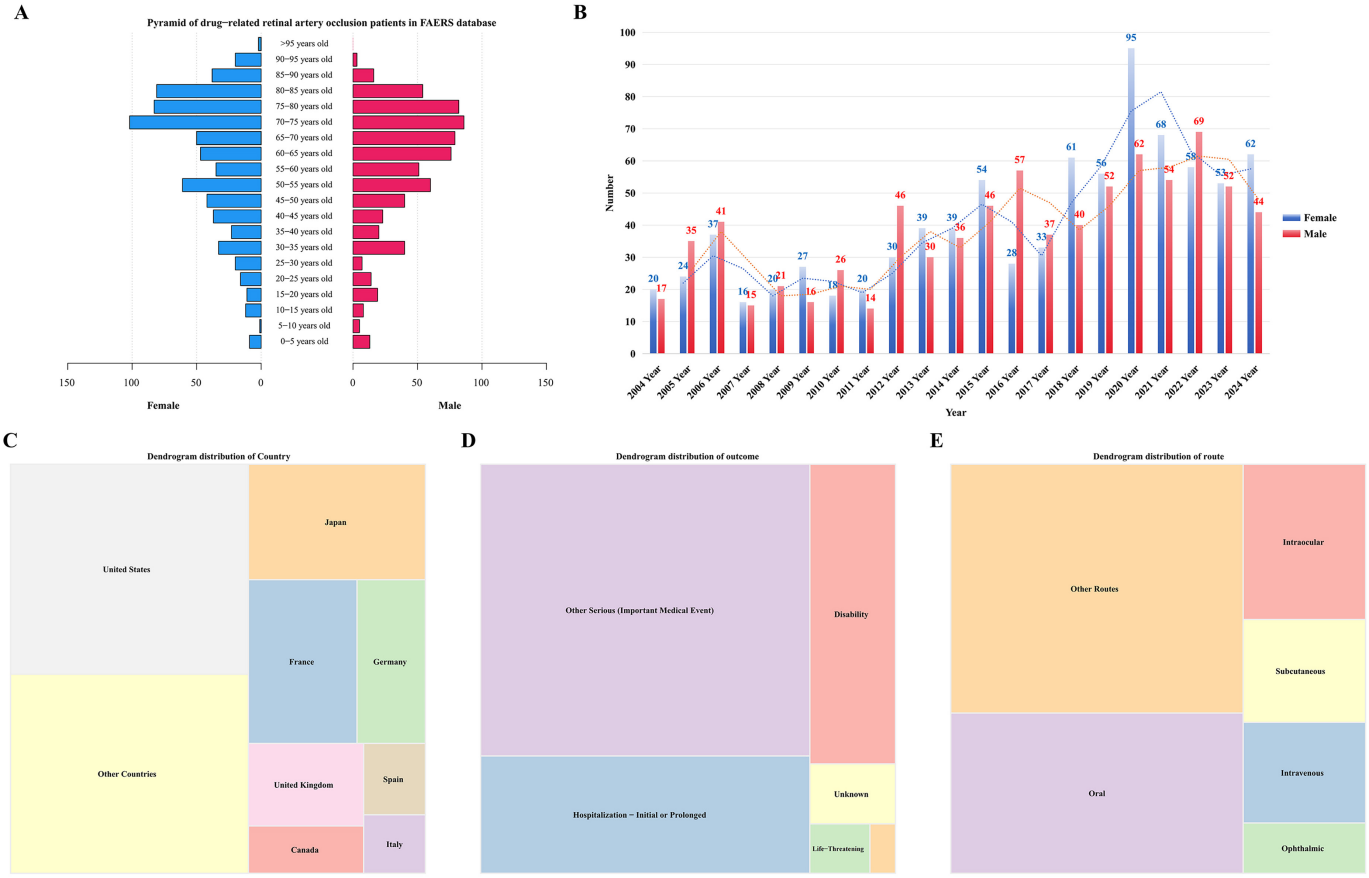
Distribution of baseline data for patients reporting adverse events of RAO in the FAERS database. **(A)** Patient age distribution by gender. **(B)** Reporting trend of adverse events. **(C)** Distribution of RAO adverse event reports by country. **(D)** Distribution of patient outcomes. **(E)** Profile of drug administration routes.

In the FAERS database, 1,927 instances of drug-related RAO adverse reactions were recorded between January 2004 to December 2024. The cohort had a mean age of 59.13 ± 20.89 years and a mean weight of 75.27 ± 20.46 kg. The study population included 858 females (44.53%) and 810 males (42.03%), with incomplete gender data for some subjects. Drug-related RAO reports were more frequently observed in older individuals, with the greatest number of reports occurring among patients aged 70–74 years (Figure 2A). Reports of drug-related RAO fluctuated over time, with the highest number reported in 2020. Overall, reporting frequencies after 2015 remained higher than those observed during the early study period. The United States, Japan, and France were the top three countries reporting these cases (Figure 2C). The most frequently reported clinical outcome associated with RAO was “other serious conditions” (55.32%; *n* = 1,066), followed by hospitalization (22.26%; *n* = 429) and disability (14.97%; *n* = 285). Less frequent outcomes included death (2.23%; *n* = 43) and life-threatening conditions (1.71%; *n* = 33) (Figure 2D). Notably, these serious outcomes may be associated with other concurrent adverse events reported, rather than directly with RAO. Of the reported RAO cases, the oral route was the most common (27.61%), followed by the intraocular route (11.21%) and subcutaneous route (7.42%) (Figure 2E). The majority of reports were submitted by physicians, accounting for 65.70% of the total, while the remaining submissions came from other healthcare professionals (19.0%), pharmacists (4.31%), and individuals from other occupations (10.9%). Further details are provided in Table 1 and Figure 2.

**TABLE 1:** Baseline data distribution of drug-related retinal artery occlusion.

Variable	Total
Age	59.13 ± 20.89
Weight	75.27 ± 20.46
Gender	
Female	858(44.53)
Male	810(42.03)
Unknown	259(13.44)
Reporter	
Physician	1266(65.70)
Other health-professional	367(19.0)
Pharmacist	83(4.31)
Unknown	211(10.9)
Year	
2004 year	37(1.92)
2005 year	59(3.06)
2006 year	78(4.05)
2007 year	33(1.71)
2008 year	46(2.39)
2009 year	45(2.34)
2010 year	50(2.59)
2011 year	41(2.13)
2012 year	89(4.62)
2013 year	74(3.84)
2014 year	83(4.31)
2015 year	113(5.86)
2016 year	100(5.19)
2017 year	93(4.83)
2018 year	110(5.71)
2019 year	124(6.43)
2020 year	181(9.39)
2021 year	158(8.20)
2022 year	155(8.04)
2023 year	130(6.75)
2024 year	128(6.64)
Country	
United States	567(29.42)
Japan	232(12.04)
France	202(10.48)
Germany	127(6.59)
United Kingdom	108(5.60)
Canada	62(3.22)
Spain	50(2.59)
Italy	41(2.13)
Other Countries	538(27.92)
Route	
Oral	532(27.61)
Intraocular	216(11.21)
Subcutaneous	143(7.42)
Intravenous	140(7.27)
Ophthalmic	70(3.63)
Other Routes	826(42.86)
Outcome	
Other Serious (Important Medical Event)	1066(55.32)
Hospitalization - Initial or Prolonged	429(22.26)
Disability	285(14.79)
Death	43(2.23)
Life-Threatening	33(1.71)
Required Intervention to Prevent Permanent Impairment/Damage	14(0.73)
Unknown	57(2.96)

### Classification of drugs associated with RAO

**FIGURE 3:**
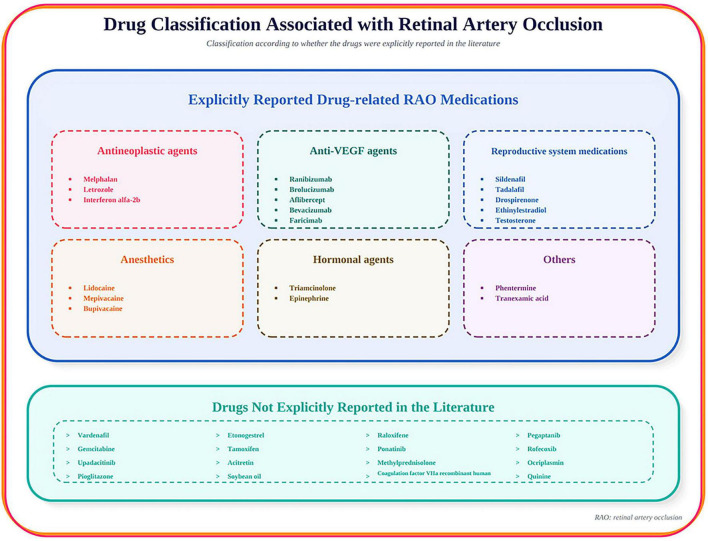
Literature-supported and newly identified drugs associated with RAO.

A total of 36 drugs with positive disproportionality signals for RAO were ultimately included in the final analysis. Among these, 20 drugs had prior literature support for an association with RAO, whereas 16 drugs were identified as signals in the present FAERS analysis without previously identified RAO reports. These findings expand the spectrum of medications potentially associated with RAO (Figure 3). The identified drugs were categorized into antineoplastic agents (8 drugs, 22.2%), anti-VEGF medications (6 drugs, 16.7%), reproductive system medications (6 drugs, 16.7%), anesthetics (3 drugs, 8.3%), anti-inflammatory drugs (3 drugs, 8.3%), hormonal agents (3 drugs, 8.3%), and other medications (7 drugs, 19.5%). Detailed information on the pharmacological characteristics and disproportionality analysis results of these drugs is provided in Supplementary Table 3.

### Disproportionality signals of drugs for RAO

**FIGURE 4:**
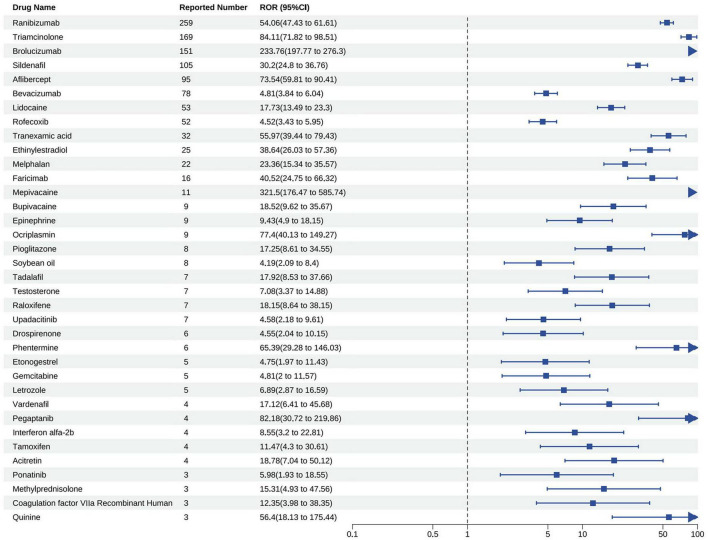
Forest plot of ROR-based positive signals for drug-related RAO from the FAERS database.

Among the analyzed drugs, ranibizumab was associated with the highest number of RAO-related reports, totaling 259 cases, followed by triamcinolone (169 cases), brolucizumab (151 cases), sildenafil (105 cases), and aflibercept (95 cases). In terms of signal strength, mepivacaine exhibited the strongest association with RAO, with an ROR of 321.5 (95% CI: 176.47–585.74). Other drugs with notably high signals included brolucizumab (ROR 233.76, 95% CI: 197.77–276.3), triamcinolone (ROR 84.11, 95% CI: 71.82–98.51), pegaptanib (ROR 82.18, 95% CI: 30.72–219.86), and ocriplasmin (ROR 77.4, 95% CI: 40.13–149.27), all of which warrant clinical attention. Additional details are provided in Figure 4 and Supplementary Table 3.

### Age- and sex-stratified analyses

**FIGURE 5:**
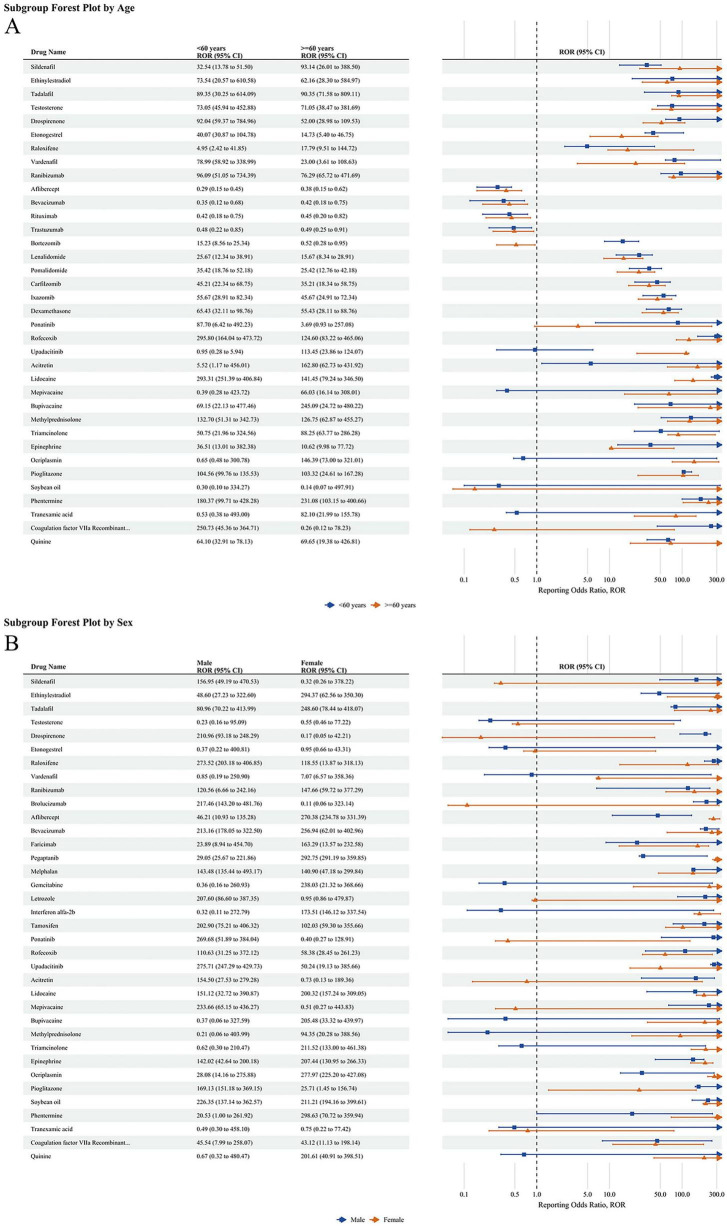
Age- and sex-stratified analyses of drug-related RAO. **(A)** Forest plot of ROR and 95% confidence intervals (CIs) for patients aged < 60 and ≥ 60 years. **(B)** Forest plot of ROR and 95% CIs for male and female patients.

Age-stratified analyses ( < 60 vs. ≥ 60 years) showed that most drug–RAO associations remained detectable within both age subgroups. Similarly, sex-stratified analyses demonstrated that most associations were observed in both male and female subgroups. These analyses were conducted to assess the presence and consistency of disproportionality signals across demographic strata, rather than to compare signal strength between groups. Detailed results are presented in Figure 5.

### Drug classification by signal strength

**FIGURE 6:**
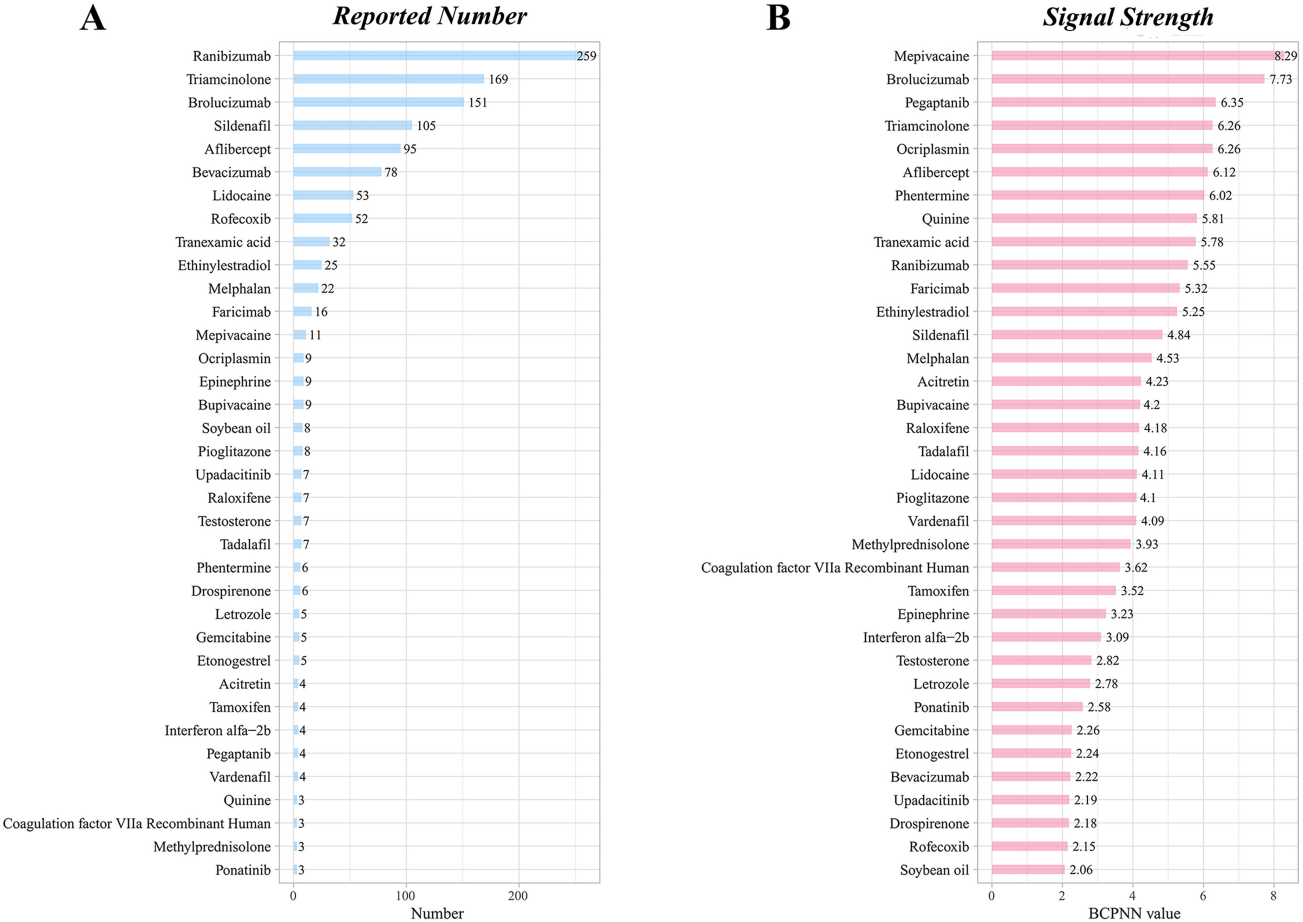
Reporting frequency and signal strength of drugs associated with RAO. **(A)** Number of RAO reports. **(B)** Signal strength based on BCPNN values.

Drug-related RAO signal strength was assessed using the BCPNN algorithm and categorized based on threshold values: weak signal (BCPNN between 0 and 1.5), moderate signal (BCPNN between 1.5 and 3), and strong signal (BCPNN above 3) (15, 16). Of the drugs assessed, 26 (72.2%) were classified as strong signal, while 10 (27.8%) were classified as moderate signal. The five drugs with the strong signal were mepivacaine (BCPNN = 8.29), brolucizumab (BCPNN = 7.73), pegaptanib (BCPNN = 6.35), triamcinolone (BCPNN = 6.26), and ocriplasmin (BCPNN = 6.26). Detailed information can be found in Figure 6.

### Drug-onset times across different pharmacological classes

**FIGURE 7:**
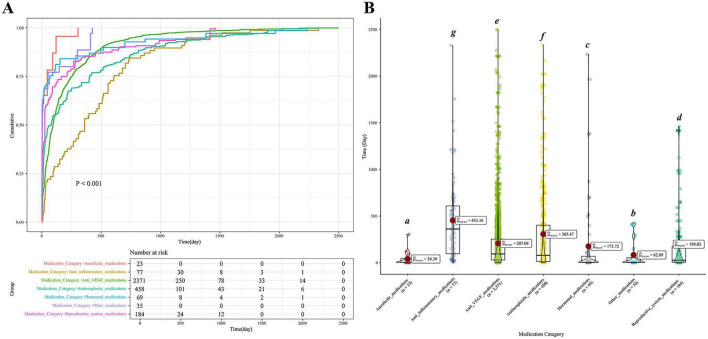
Onset time of adverse reactions in drug-related RAO. **(A)** Analysis of cumulative reports of drug-related RAO across drug classes over time. **(B)** Median onset times for drug-related RAO by drug classification.

To evaluate differences in drug-induced onset times, medications were categorized, and cumulative risk curves were compared. The analysis revealed a significant disparity in onset times across categories. Anesthetic medications had the shortest onset time, with a mean of 39.39 days, while anti-inflammatory medications demonstrated the longest onset time, with a mean of 453.16 days. These results are visually summarized in Figure 7.

## Discussion

To our knowledge, this is one of the first comprehensive pharmacovigilance studies to systematically evaluate drug–RAO associations across multiple therapeutic classes using the FAERS database. By integrating four complementary disproportionality algorithms, we identified 36 drugs with positive reporting signals, including 20 drugs previously described in the literature and 16 drugs not previously reported in association with RAO. These findings broaden the spectrum of medications potentially associated with RAO and generate hypotheses for future epidemiological and mechanistic investigations.

A notable upward trend in reports of drug-related RAO over the past 5 years may reflect increased drug utilization, greater awareness of adverse event reporting systems, and advancements in diagnostic imaging. This may reflect increasing recognition and reporting of drug-related RAO. Additionally, our analysis showed a higher reporting frequency of drug-related RAO among older individuals, with the greatest number of reports observed in patients aged 70–74 years, consistent with the known epidemiological characteristics of RAO (17). Because age and sex may influence reporting patterns, stratified analyses were performed as exploratory assessments. However, these analyses were not intended for formal comparisons between demographic groups. Consequently, any apparent differences across subgroups should be interpreted cautiously and require confirmation in future studies.

Anti-VEGF medications were associated with RAO, with previous reports documenting retinal artery occlusion after intravitreal ranibizumab, aflibercept, bevacizumab, brolucizumab, and faricimab injections (18, 19). Among these drugs, ranibizumab accounted for the highest number of reported cases. Ranibizumab is widely used and approved for various retinal conditions, including neovascular age-related macular degeneration (nAMD), diabetic macular edema, macular edema secondary to retinal vein occlusion, and myopic choroidal neovascularization (20). Notably, brolucizumab showed the most pronounced disproportionality signal, with an exceptionally high ROR of 233.76 (95% CI: 197.77–276.30), indicating a particularly strong disproportionality signal with RAO among anti-VEGF agents. Brolucizumab, a novel anti-VEGF therapeutic, represents a significant advancement in treating nAMD (21).However, the present FAERS-based analysis cannot distinguish whether the observed RAO signals are attributable to the pharmacological effects of anti-VEGF agents, the intravitreal injection procedure itself, procedure-related complications, or underlying patient comorbidities. Intravitreal injections may independently contribute to retinal vascular events through transient intraocular pressure elevation, local vascular compression, or injection-related embolic phenomena (22, 23). Therefore, the detected disproportionality signals should be interpreted cautiously and not considered evidence of a direct causal relationship. Further prospective and mechanistic studies are required to clarify the relative contributions of drug-related and procedure-related factors.

In this study, reproductive system medications represented a substantial proportion of the identified RAO signals, primarily involving sex hormone agents and phosphodiesterase type 5 (PDE5) inhibitors. Among these drugs, ethinylestradiol, a synthetic estrogen widely used in oral contraceptives and hormone replacement therapy, demonstrated a notably strong disproportionality signal (ROR = 38.64). Oral contraceptives have been implicated in RAO through their prothrombotic effects on the coagulation system. A previous case report described a 22-year-old woman who developed RAO after receiving a low-dose ethinylestradiol-containing contraceptive, accompanied by elevated thrombin–antithrombin complex levels and reduced free protein S activity, suggesting a hypercoagulable state as a potential mechanism (24). Similar retinal arterial occlusive events have also been reported in patients receiving testosterone therapy, further supporting the role of hormone-related coagulation disturbances in the pathogenesis of RAO (25). In addition, significant pharmacovigilance signals were observed for the PDE5 inhibitors sildenafil (ROR = 30.20), tadalafil (ROR = 17.92), and vardenafil (ROR = 17.12). Previous case reports have documented retinal artery occlusion following sildenafil and tadalafil use, supporting the biological plausibility of the associations identified in the present study (26, 27). Although the precise mechanism remains unclear, several hypotheses have been proposed. PDE5 inhibition enhances nitric oxide–cyclic guanosine monophosphate (NO–cGMP) signaling, which may alter retinal vascular autoregulation and blood flow dynamics. These hemodynamic changes could contribute to retinal ischemia and increase susceptibility to arterial occlusive events in predisposed individuals (28).

Among anesthetic drugs, mepivacaine showed a particularly strong signal (ROR = 321.50), while lidocaine (ROR = 17.73) and bupivacaine (ROR = 18.52) also exhibited significant positive signals. In our FAERS analysis, anesthetic agents demonstrated the shortest mean time to onset among all drug classes (39.39 days). Literature evidence indicates that RAO associated with ocular or periocular anesthesia—most commonly after regional blocks such as retrobulbar or peribulbar injections—typically presents acutely or subacutely, often recognized on the day of surgery or within the first postoperative day, with a smaller subset occurring up to several weeks later (29). The discrepancy between published case reports and FAERS-derived TTO may reflect delayed reporting, missing onset dates, or heterogeneous clinical settings captured in spontaneous reporting systems. Proposed mechanisms for anesthetic-associated RAO are multifactorial. Pianka et al. (30) and Findl et al. (31) demonstrated that local anesthetic solutions can reduce ophthalmic artery blood flow through vasoconstriction. Morgan et al. (32) suggested that the injection procedure may induce traumatic spasms of the central retinal artery in susceptible individuals. Additional contributors include inadvertent intra-arterial injection, embolic phenomena, optic nerve sheath compression, and transient or sustained intraocular pressure elevation (29). Given the strong disproportionality signals and potential visual consequences, clinicians performing retrobulbar, peribulbar, or other orbital blocks should remain vigilant, particularly in patients with pre-existing vascular risk factors.

Time-to-onset (TTO) data derived from spontaneous reporting systems are frequently affected by incomplete or missing dates, which may limit the precision of latency estimates. Nevertheless, distinct onset patterns were observed across drug classes. While anesthetic-associated RAO typically occurred shortly after exposure, consistent with previous reports describing acute retinal arterial occlusion following regional ophthalmic anesthesia (29), anti-inflammatory medications were characterized by substantially longer latency periods, often following prolonged treatment. This pattern may reflect cumulative vascular injury or thrombotic effects associated with long-term exposure to certain anti-inflammatory and corticosteroid therapies (33, 34). However, the prolonged TTO observed for anti-inflammatory medications has not been specifically established in the literature and should be interpreted cautiously given the limitations of spontaneous reporting data.

Among the identified agents, melphalan, tamoxifen, and interferon alfa-2b demonstrated particularly strong disproportionality signals. Previous studies have linked melphalan to retinal and ophthalmic arterial occlusive events after intra-arterial chemotherapy for retinoblastoma, whereas tamoxifen and interferon alfa-2b have been associated with thromboembolic and retinal vascular complications, respectively (10, 35–37). These findings support the biological plausibility of the signals detected in the present analysis. In addition to these previously recognized agents, significant disproportionality signals were observed for gemcitabine, letrozole, and ponatinib. Although direct evidence linking these drugs to RAO remains limited, vascular toxicity and thromboembolic complications have been reported with each of these therapies. In particular, ponatinib is known to increase the risk of arterial occlusive events through endothelial dysfunction and accelerated atherosclerotic changes, providing biological plausibility for the strong signal observed in our analysis. Collectively, these findings suggest that vascular injury and thrombosis may represent common mechanisms underlying the association between antineoplastic therapies and RAO. Given the increasing use of targeted therapies and the prolonged survival of patients with malignancies, clinicians should remain aware of potential ocular vascular adverse events, especially in individuals with pre-existing cardiovascular risk factors. However, the observed signals should be interpreted cautiously, as disproportionality analyses cannot establish causality and require confirmation in future epidemiological studies.

Regarding anti-inflammatory and hormonal medications, several agents demonstrated notable disproportionality signals for RAO. Among anti-inflammatory drugs, acitretin has been associated with vascular and thrombotic adverse events, providing biological plausibility for the observed signal. For hormonal medications, corticosteroid-related agents generated notable reporting signals. Previous studies have documented retinal arterial occlusive events following intravitreal or periocular corticosteroid administration, supporting a potential association between corticosteroid exposure and retinal vascular compromise (38, 39). Although the precise mechanisms remain unclear, corticosteroid-induced alterations in vascular perfusion, endothelial function, and coagulation pathways have been proposed as potential contributors to retinal ischemia. These observations warrant further investigation in future studies.

Beyond the major pharmacological classes, several other medications were identified in our analysis with significant disproportionality signals for RAO. These drugs, diverse in their primary indications and mechanisms of action, collectively highlight a potential involvement in RAO reporting signals. For instance, the notably high ROR for tranexamic acid (ROR = 55.97), an antifibrinolytic agent, can be mechanistically explained by its ability to promote spontaneous thrombosis, particularly in individuals with predisposing factors (40). Similarly, phentermine, an appetite suppressant used for short-term obesity management, also showed strong signals, potentially due to drug-induced vasoconstriction or vasospasm, which could lead to retinal ischemia (41). Additionally, ocriplasmin and quinine demonstrated strong signals. Other agents, such as pioglitazone, soybean oil, and coagulation factor VIIa, further expanded the spectrum of drugs associated with RAO risk. The inclusion of these varied agents highlights that the risk of RAO is not confined to a few specific drug classes, but can be linked to a broader range of medications. Consequently, this research provides valuable insights into drugs potentially associated with RAO and supports the need for future in-depth investigations into their risk mechanisms.

This study has several limitations inherent to the analysis of spontaneous reporting systems. First, the identified signals indicate statistical associations but cannot establish definitive causal relationships between the drugs and RAO. Second, the presence of reporting biases, such as over-reporting for novel drugs (e.g., brolucizumab) or under-reporting of well-established events, may influence the magnitude of the disproportionality signals. Third, the lack of detailed clinical information, including patients’ baseline comorbidities, concomitant medications, and precise ocular findings, precludes a thorough assessment of potential confounding factors and underlying mechanisms. Furthermore, the absence of denominator data (i.e., the total number of patients exposed to each drug) prevents the calculation of true incidence rates. Consequently, while this analysis highlights important potential associations, the findings should be interpreted as generating hypotheses rather than confirming drug-related risks, and they do not support the development of a stratified clinical monitoring framework. Future studies integrating multisource data, such as electronic health records, are warranted to validate these associations and elucidate the clinical risk factors.

## Conclusion

Using the FAERS database, this study identified and characterized drugs with disproportionality signals associated with retinal artery occlusion (RAO). The findings provide an overview of reporting patterns, the strength of statistical associations, and onset characteristics of drug-related RAO, and may contribute to improved awareness of this potentially vision-threatening adverse event. Given the inherent limitations of spontaneous reporting systems and the absence of Weibull distribution modeling for time-to-onset analysis, these findings should be considered hypothesis-generating and interpreted with caution. Further epidemiological, prospective, and mechanistic studies are required to validate the observed associations.

## Data Availability

Publicly available datasets were analyzed in this study. This data can be found at: https://fis.fda.gov/extensions/FPD-QDE-FAERS/FPD-QDE-FAERS.html.

## Footnotes

1
https://fis.fda.gov/extensions/FPD-QDE-FAERS/FPD-QDE-FAERS.html

2
https://go.drugbank.com/
