# Supplementary material for: Signals of drug-related retinal artery occlusion: a multi-country retrospective study from a spontaneous reporting system
Source: Front Med (Lausanne). 2026 Jul 7;13:1851758. doi: 10.3389/fmed.2026.1851758 (PMC13386543; doi:10.3389/fmed.2026.1851758)
Supplement: Supplementary file 1 [file Table_1.docx]

| **Supplementary Table 1. Four-grid table of disproportionality analysis method.** | | | |
| --- | --- | --- | --- |
| Item | Target adverse events | All other adverse events | Total |
| Target drugs | a | b | a+b |
| All other drugs | c | d | c + d |
| Total | a+c | b+d | a+b + c + d |

**Notes:** A contingency table for the calculation formula of the proportion imbalance analysis.
