# Supplementary material for: Signals of drug-related retinal artery occlusion: a multi-country retrospective study from a spontaneous reporting system
Source: Front Med (Lausanne). 2026 Jul 7;13:1851758. doi: 10.3389/fmed.2026.1851758 (PMC13386543; doi:10.3389/fmed.2026.1851758)
Supplement: Supplementary file 2 [file Table_2.docx]

| **Supplementary Table 2. Principle of disproportionality analysis and standard of signal detection.** | | |
| --- | --- | --- |
| **Methods** | **Calculation formula** | **Inclusion standard of positive signal** |
| ROR | $ROR =\frac{(a/c)}{(b/d)}$ | a≥3 and lower limit of 95% CI > 1 |
|  | $SE(\ln\mathrm{ROR})=\sqrt{(\frac{1}{a}+\frac{1}{b}+\frac{1}{c}+\frac{1}{d})}$ |  |
|  | $95\%CI = e^{\ln(ROR)\pm1.96\sqrt{\frac{1}{a}+\frac{1}{b}+\frac{1}{c}+\frac{1}{d}}}$ |  |
| PRR | $PRR =\frac{a/(a+b)}{c/(c+d)}$ | a≥3 and lower limit of 95% CI > 1 |
|  | $SE(\ln\mathrm{PRR})=\sqrt{(\frac{1}{a}-\frac{1}{a+b}+\frac{1}{c}-\frac{1}{c+d})}$ |  |
|  | $95\%CI = e^{\ln(PRR)\pm1.96\sqrt{(\frac{1}{a}-\frac{1}{a+b}+\frac{1}{c}-\frac{1}{c+d})}}$ |  |
|  | $X^{2}=\frac{{（ad-bc）}^{2} \times(a+b+c+d)}{(a+b)(a+c)(b+d)(c+d)}$ |  |
| BCPNN | $IC = \log_{2} \frac{a(a+b+c+d)}{(a+b)(a+c)}$ | 1. No Signal(-): IC_025_≤0 2. Low Signal(+):0<IC_025_≤1.5 3. Medium Signal(++):1.5<IC_025_≤3 4. High Signal(+++): IC_025_>3 |
|  | $\mathrm{IC}_{025} = \log_{2} \frac{(a+\gamma11)(a+b+c+d+\alpha)(a+b+c+d+\beta)}{(a+b+c+d+\gamma)(a+b+\alpha1)(a+c+\beta1)}$ |  |
|  | $V(IC)=\frac{1}{{(\ln2)}^{2}}\{[\frac{(a+b+c+d)-a+\gamma-\gamma11}{(a+\gamma11)(1+a+b+c+d+\gamma)}]+[\frac{(a+b+c+d)-(a+b)+a-\alpha1}{(a+b+\alpha1)(1+a+b+c+d+\alpha)}]+[\frac{(a+b+c+d)-(a+c)+\beta-\beta1}{(a+c+\beta1)(1+a+b+c+d+\beta)}]\}$ |  |
|  | $\gamma= \gamma11\frac{(a+b+c+d+\alpha)(a+b+c+d+\beta)}{(a+b+\alpha1)(a+c+\beta1)}$ |  |
|  | $IC-2SD = E(IC)-2\sqrt{V(IC)}$ |  |
|  | Where α1 = β1 =1; α = β =2; $\gamma11 = 1$ |  |
| MGPS | $EBGM=\frac{a(a+b+c+d)}{(a+c)(a+b)}$ | EBGM05>2 and a>0 |
|  | $EBGM05=e^{\ln(EBGM)-\sqrt[2]{1.64(\frac{1}{a}+\frac{1}{b}+\frac{1}{c}+\frac{1}{d})}}$ |  |

**Abbreviation:** ROR, reporting odds ratio; PRR, proportional reported ratio; BCPNN, Bayesian confidence propagation neural network; MGPS, multi-item gamma poisson shrinker; CI, confidence interval; IC, information component.
