# Supplementary material for: Signals of drug-related retinal artery occlusion: a multi-country retrospective study from a spontaneous reporting system
Source: Front Med (Lausanne). 2026 Jul 7;13:1851758. doi: 10.3389/fmed.2026.1851758 (PMC13386543; doi:10.3389/fmed.2026.1851758)
Supplement: Supplementary file 3 [file Table_3.docx]

**Supplementary Table 3. Disproportionality Analysis Results of Drug-Related Retinal Artery Occlusion**

| **Drug Name** | **Drug Classfication** | **ROR (95%CI)** | **PRR (95%CI)** | **MGPS (95%CI)** | **BCPNN (95%CI)** | **PRR (X2)** | **P value** |
| --- | --- | --- | --- | --- | --- | --- | --- |
| Sildenafil | Reproductive system medications | 30.2(24.8 to 36.76) | 30.13(29.93 to 30.33) | 28.6(24.26 to 33.71) | 4.84(3.17 to 6.5) | 30.13(2801.67) | <0.001 |
| Ethinylestradiol | Reproductive system medications | 38.64(26.03 to 57.36) | 38.53(38.14 to 38.92) | 38.06(27.35 to 52.97) | 5.25(3.58 to 6.92) | 38.53(902.5) | <0.001 |
| Tadalafil | Reproductive system medications | 17.92(8.53 to 37.66) | 17.9(17.15 to 18.64) | 17.84(9.58 to 33.2) | 4.16(2.49 to 5.82) | 17.9(111.28) | <0.001 |
| Testosterone | Reproductive system medications | 7.08(3.37 to 14.88) | 7.08(6.34 to 7.82) | 7.06(3.79 to 13.14) | 2.82(1.15 to 4.49) | 7.08(36.43) | <0.001 |
| Drospirenone | Reproductive system medications | 4.55(2.04 to 10.15) | 4.55(3.75 to 5.35) | 4.54(2.32 to 8.88) | 2.18(0.52 to 3.85) | 4.55(16.58) | <0.001 |
| Etonogestrel | Reproductive system medications | 4.75(1.97 to 11.43) | 4.75(3.87 to 5.63) | 4.74(2.27 to 9.88) | 2.24(0.58 to 3.91) | 4.75(14.76) | 0.001 |
| Raloxifene | Reproductive system medications | 18.15(8.64 to 38.15) | 18.13(17.39 to 18.87) | 18.07(9.71 to 33.63) | 4.18(2.51 to 5.84) | 18.13(112.9) | <0.001 |
| Vardenafil | Reproductive system medications | 17.12(6.41 to 45.68) | 17.09(16.11 to 18.07) | 17.06(7.5 to 38.79) | 4.09(2.42 to 5.76) | 17.09(60.49) | <0.001 |
| Ranibizumab | Anti-VEGF medications | 54.06(47.43 to 61.61) | 53.86(53.73 to 53.99) | 47(42.12 to 52.43) | 5.55(3.89 to 7.22) | 53.86(11692.52) | <0.001 |
| Brolucizumab | Anti-VEGF medications | 233.76(197.77 to 276.3) | 229.92(229.76 to 230.09) | 212.59(184.83 to 244.51) | 7.73(6.06 to 9.4) | 229.92(31812.94) | <0.001 |
| Aflibercept | Anti-VEGF medications | 73.54(59.81 to 90.41) | 73.15(72.94 to 73.35) | 69.71(58.64 to 82.86) | 6.12(4.46 to 7.79) | 73.15(6438.52) | <0.001 |
| Bevacizumab | Anti-VEGF medications | 4.81(3.84 to 6.04) | 4.81(4.59 to 5.04) | 4.66(3.86 to 5.64) | 2.22(0.55 to 3.89) | 4.81(226.46) | <0.001 |
| Faricimab | Anti-VEGF medications | 40.52(24.75 to 66.32) | 40.39(39.9 to 40.89) | 40.08(26.54 to 60.53) | 5.32(3.66 to 6.99) | 40.39(609.82) | <0.001 |
| Pegaptanib | Anti-VEGF medications | 82.18(30.72 to 219.86) | 81.67(80.69 to 82.64) | 81.5(35.77 to 185.69) | 6.35(4.68 to 8.02) | 81.67(318.1) | <0.001 |
| Melphalan | Antineoplastic medications | 23.36(15.34 to 35.57) | 23.31(22.9 to 23.73) | 23.07(16.23 to 32.8) | 4.53(2.86 to 6.2) | 23.31(464.73) | <0.001 |
| Gemcitabine | Antineoplastic medications | 4.81(2 to 11.57) | 4.81(3.93 to 5.69) | 4.8(2.3 to 10) | 2.26(0.6 to 3.93) | 4.81(15.05) | 0.001 |
| Letrozole | Antineoplastic medications | 6.89(2.87 to 16.59) | 6.89(6.01 to 7.77) | 6.88(3.3 to 14.34) | 2.78(1.11 to 4.45) | 6.89(25.12) | <0.001 |
| Interferon alfa-2b | Antineoplastic medications | 8.55(3.2 to 22.81) | 8.54(7.56 to 9.52) | 8.53(3.75 to 19.38) | 3.09(1.42 to 4.76) | 8.54(26.59) | <0.001 |
| Tamoxifen | Antineoplastic medications | 11.47(4.3 to 30.61) | 11.46(10.48 to 12.44) | 11.44(5.03 to 26.01) | 3.52(1.85 to 5.18) | 11.46(38.13) | <0.001 |
| Ponatinib | Antineoplastic medications | 5.98(1.93 to 18.55) | 5.97(4.84 to 7.11) | 5.97(2.31 to 15.4) | 2.58(0.91 to 4.24) | 5.97(12.41) | 0.005 |
| Rofecoxib | Anti inflammatory medications | 4.52(3.43 to 5.95) | 4.52(4.24 to 4.79) | 4.43(3.52 to 5.57) | 2.15(0.48 to 3.81) | 4.52(138.74) | <0.001 |
| Upadacitinib | Anti inflammatory medications | 4.58(2.18 to 9.61) | 4.57(3.83 to 5.32) | 4.56(2.45 to 8.49) | 2.19(0.52 to 3.86) | 4.57(19.48) | <0.001 |
| Acitretin | Anti inflammatory medications | 18.78(7.04 to 50.12) | 18.75(17.77 to 19.73) | 18.72(8.23 to 42.56) | 4.23(2.56 to 5.89) | 18.75(67.1) | <0.001 |
| Lidocaine | Anesthetic medications | 17.73(13.49 to 23.3) | 17.71(17.44 to 17.98) | 17.26(13.74 to 21.7) | 4.11(2.44 to 5.78) | 17.71(813.42) | <0.001 |
| Mepivacaine | Anesthetic medications | 321.5(176.47 to 585.74) | 313.74(313.15 to 314.32) | 312.01(188.88 to 515.42) | 8.29(6.61 to 9.96) | 313.74(3410.5) | <0.001 |
| Bupivacaine | Anesthetic medications | 18.52(9.62 to 35.67) | 18.5(17.84 to 19.15) | 18.42(10.65 to 31.87) | 4.2(2.54 to 5.87) | 18.5(148.32) | <0.001 |
| Methylprednisolone | Hormonal medications | 15.31(4.93 to 47.56) | 15.3(14.17 to 16.43) | 15.28(5.92 to 39.43) | 3.93(2.27 to 5.6) | 15.3(40.03) | <0.001 |
| Triamcinolone | Hormonal medications | 84.11(71.82 to 98.51) | 83.62(83.46 to 83.78) | 76.62(67.13 to 87.45) | 6.26(4.59 to 7.93) | 83.62(12627.5) | <0.001 |
| Epinephrine | Hormonal medications | 9.43(4.9 to 18.15) | 9.42(8.77 to 10.08) | 9.38(5.42 to 16.23) | 3.23(1.56 to 4.9) | 9.42(67.44) | <0.001 |
| Ocriplasmin | Other medications | 77.4(40.13 to 149.27) | 76.94(76.29 to 77.6) | 76.6(44.22 to 132.71) | 6.26(4.59 to 7.93) | 76.94(671.63) | <0.001 |
| Pioglitazone | Other medications | 17.25(8.61 to 34.55) | 17.23(16.53 to 17.92) | 17.16(9.6 to 30.69) | 4.1(2.43 to 5.77) | 17.23(121.8) | <0.001 |
| Soybean oil | Other medications | 4.19(2.09 to 8.4) | 4.19(3.5 to 4.89) | 4.18(2.34 to 7.47) | 2.06(0.4 to 3.73) | 4.19(19.37) | <0.001 |
| Phentermine | Other medications | 65.39(29.28 to 146.03) | 65.07(64.27 to 65.87) | 64.87(33.12 to 127.06) | 6.02(4.35 to 7.69) | 65.07(377.39) | <0.001 |
| Tranexamic acid | Other medications | 55.97(39.44 to 79.43) | 55.74(55.39 to 56.08) | 54.86(40.93 to 73.52) | 5.78(4.11 to 7.45) | 55.74(1692.63) | <0.001 |
| Coagulation factor VIIa Recombinant Human | Other medications | 12.35(3.98 to 38.35) | 12.34(11.21 to 13.47) | 12.32(4.78 to 31.8) | 3.62(1.96 to 5.29) | 12.34(31.22) | <0.001 |
| Quinine | Other medications | 56.4(18.13 to 175.44) | 56.15(55.02 to 57.28) | 56.07(21.69 to 144.93) | 5.81(4.14 to 7.48) | 56.15(162.28) | <0.001 |
|  |  |  |  |  |  |  |  |

Note: The p-value is the statistical test result from the chi-square test used in the PRR algorithm. Each of the drugs mentioned satisfies the positive signal screening criteria for disproportionality analysis.

Abbreviations: BCPNN, Bayesian confidence propagation neural network; MGPS, multiitem gamma Poisson shrinker; PRR, proportional reported ratio; ROR, reporting odds ratio; CI, confidence interval.
